# The Enemy of the Race

**Published:** 1918-03-09

**Authors:** 


### The Hospital

**The Workers' Newspaper of Administrative Medicine and Institutional Life, Administration, National Insurance and Health.**

No. 1657, Vol. LXIII. SATURDAY, MARCH 9, 1918. PRICE ONE PENNY

### THE ENEMY OF THE RACE.

The war has now lasted three years and a half: it has taken a dreadful toll of millions of lives, and has produced an amount of suffering and misery altogether incalculable. Yet the war has not killed so many people as the numbers of those who, in the belligerent countries, are infected with venereal disease, and it may be doubted whether the amount of misery produced by wounds and death in the battles of any one year equals that produced in the same year by venereal disease. The number of British subjects killed and wounded in the war up to the present moment is, perhaps, not accurately known, except to the authorities, but it is certainly not equal to one-tenth of the whole population of these islands. It is certainly not five millions. It is certainly not the half of five millions; but at the present time five millions of the inhabitants of this country are suffering from venereal disease in one form or another.

The war, if it continues long enough, may decimate the existing population. Venereal disease has already virtually decimated the population, by preventing births, or by causing abortion, still-births, and infantile mortality. The war kills the young, the vigorous, and the strong; but at least they know the danger they incur, and when they go into the war zone they go with their eyes open to the possible result. They are not betrayed to suffering and death by their nearest and dearest. This is, however, too often the way in which innocent women, aye, and innocent men also, are betrayed and infected with venereal disease.

We are led into these reflections by the account of a public meeting held last month in the Free Trade Hall in Manchester, under the presidency of the Lord Mayor, and attracting an audience which almost filled the Hall. As the Free Trade Hall accommodates 4,000 people, this indicates that public opinion, at any rate in Manchester, is at long last being aroused and cultivated on this immensely important subject. "There have been considerations," said the Lord Mayor, Sir Alexander Porter, "both of religion and morals, that have prevented the subject from having proper consideration; but the public has become awakened." We know not what religion has to say to venereal disease, and as to morals, what has prevented the full and proper discussion of the subject has been, not morals, but a sentimental and fastidious squeamishness, a moral cowardice that has prevented the public from looking the facts steadily in the face. In this matter the medical profession has been to blame, for it alone knew the full extent of the evil. Now the evil is known, but it is not known widely enough, and it is not reckoned with seriously enough. At the same meeting, Mr. Hayes Fisher, M.P., President of the Local Government Board, truly said that "Taken in the aggregate for the last hundred years, venereal disease had caused more pain and misery, broken up more homes, sapped more of the vitality of the nation, rendered more women sterile, and had done more harm and hurt than all that had been done by the foul weapons employed against us in this great war. It is the enemy of every man, woman, and child, and should be treated as such." Mr. Hayes Fisher spoke well within the mark—too much so. He did not put his points nearly strongly enough. If he had substituted twenty years for one hundred he would still have been within the mark. He would still have understated his case.

Sir Thomas Barlow told the meeting that the greatest mortality from these diseases is amongst casual labourers and the class immediately above them, but next on the list come the upper and middle classes, and only after these the skilled artisans, while agricultural labourers suffer least of all. With respect to the incidence of the disease, two facts stand out prominently, and can never be too often or too much insisted on: first, that no class in the whole community, from the highest to the lowest, is exempt; and, second, that venereal disease is by no means necessarily associated with immorality in the sufferer. The last is especially important, since, although it is well enough known to the medical profession, it is scarcely at all known to the laity. It cannot be proclaimed too often, too far, or too widely, that great multitudes of chaste and innocent women are rendered sterile for life by venereal disease contracted from their husbands in the early days of marriage; disease whose nature the wife never knows, and from which the husband has long since believed himself cured. There is a silly and pernicious notion abroad that venereal disease is necessarily due to immorality, and that to oppose or to prevent the disease is an unjustifiable interference with the just punishment that the Almighty visits upon sin. Nothing could be more false; nothing could be more pernicious. Multitudes of women who have married in the confident hope of becoming joyful mothers of children have had this hope for ever blasted by a mysterious cause of whose nature they are ignorant. Not only so, but the childless woman may be held responsible for this misfortune. A case, an example of many, has recently come to our knowledge, in which a woman had been reproached by her husband and her husband's family with barrenness. She submitted to examination at the hands of a gynæcologist, who could discover no reason why she should not bear children. It occurred to him then to examine the husband, and the husband was found to be the subject of long-standing and doubtless obliterating epididymitis, the result of a long-past gonorrhœa. Such are the ramifications, such the consequences, of venereal disease.

Since this article was written a most notable thing has happened. His Majesty the King has, with the absence of ostentation with which he does so many good works, but publicly and openly, visited the Rochester Row Hospital, and has issued from Buckingham Palace a notification that he has done so. Who will pretend after this that venereal diseases do not exist, or that if they do, it is not respectable to talk of them?

